# Supplementary material for: Inflammatory cytokines, placental pathology, and neurological outcomes in infants born to preterm preeclamptic mothers
Source: PLoS One. 2021 Nov 15;16(11):e0260094. doi: 10.1371/journal.pone.0260094 (PMC8592443; doi:10.1371/journal.pone.0260094)
Supplement: S1 File — (PDF) [file pone.0260094.s001.pdf]

PAEDIATRIC STROKE OUTCOME MEASURE SHORT NEURO EXAM (PSOM-SNE) INFANT VERSION (INFANTS TERM BIRTH TO 2 YEARS)

IDENTIFYING DATA

IPSS ID# _______________ **Site:** ____________________ **Date of assessment** (yyyy-mm-dd): ___________________________

Type of Assessment: ☐ Initial visit ☐ Follow-up **Location of Assessment:** ☐ In-patient ☐ Clinic

INSTRUCTIONS: Check appropriate column for each item: Abnormal; Normal or Not Done (includes not age appropriate item)

LEVEL OF CONSCIOUSNESS

TEST ITEM	Normal	Abnormal	Notes
Level Of Consciousness			

BEHAVIOUR, MENTAL STATUS

TEST ITEMS	Not Done	Normal	Abnormal	Guidelines for Scoring
Activity Level				Excessively quiet, shy, removed, Hyperactive, fidgety
Interpersonal Interaction				With parents and examiner
Cooperation				Age dependent
Attention				
Affect				Extremely shy, withdrawn totally flat, gaze avoidance, hyperactive
Object Permanence				Test Ages 4–12 Months. Should be present by 8 Months

LANGUAGE

TEST ITEMS	Not Done	Normal	Abnormal	Guidelines for Scoring
Language Development				**Normal:**<br>**0-4 mos**.- Coos **4-12 mos**. - babbles<br>**by 12 mos**. - 1-2 words **12-18 mos**. - single words<br>**2 years**. - 2 word phrase **3 years** - 3 word sentence, 200 words

CRANIAL NERVES

TEST ITEMS		Not Done	Normal	Abnormal	Guidelines for Scoring and Notes (Describe Abnormalities)
Visual Fields / Vision	Right				Facing patient at 2–3 ft encourage to stare at your eyes and tell when they see object come into view from side (or note gaze shifting toward object)
	Left				
Pupillary Light Reflex	Right				Direct and Consensual
	Left				
Funduscopy	Right				Note Abnormalities:
	Left				
Ocular Motility	Right				Move pen or red object or light smoothly from right to left and back testing full range. Watch for nystagmus or dysconjugate eye movements
	Left				
Optokinetic Nystagmus	Right				Test from 6 mos: move measuring tape slowly from right to left and back through full range encourage to 'look at the lines'
	Left				
Facial Sensation	Right				Touch each side with light touch and cold object comparing forehead, cheek and chin R / L: watch for child's reaction
	Left				
Facial Movements	Right				Observe smile, observe mouth symmetry during vocalization. Listen to speech quality, observe eye closure for symmetry
	Left				
Hearing	Right				Finger-rub for infants or whisper at 2 - 3 feet away.
	Left				
Swallow					
Palate and gag	Right				Observe during open mouth crying or demonstrate with tongue protruded 'Say 'ahhhhh.' Listen to voice quality
	Left				
Trapezius Strength	Right				Test Shoulder Shrug
	Left				
Tongue Movements Side-To-Side	Right				
	Left				

PSOM: Pediatric Stroke Outcome Measure-Neuro. Exam. Children's Stroke Program, Hospital for Sick Children, Toronto, Canada.
G. deVeber, D. MacGregor, R. Curtis, T. Soman, R. Ichord et al. Version October 2003, format revised November 2005

INFANT GROSS MOTOR

Infant Primitive Reflexes (Test Only Infants <12 months of age)

TEST ITEMS		Not Done	Normal	Abnormal	Guidelines for Scoring
Sucking Reflex					
Rooting Reflex					
Palmer Grasp Reflex	Right				$\leq$ 4 months
	Left				
Plantar Grasp Reflex	Right				$\leq$ 12 months
	Left				
Stepping and Placing Reflexes	Right				$\leq$ 6 months
	Left				
Asymmetric Tonic Neck Reflex					$\leq$ 6 months
Moro Reflex	Right				$\leq$ 6 months
	Left				
Parachute Reflex	Right				**Symmetry Norms:**<br>Downward By $\leq$ 6 months<br>Sideways By $\leq$ 8 months<br>Forward By 12 months
	Left				

Developmental Gross Motor

Test Only: Infants <2 Yrs OR Children $\geq$2 Yrs without Independent Ambulatory Motor Function

TEST ITEMS	Not Done	Normal	Abnormal	Guidelines for Scoring
Central Tone: Head lag on 'pull-to-sit'				
Central Tone: Slip Thru On Vertical Suspension				
Central Tone: Tone on Ventral Suspension				
Rolls Over (Front To Back)				By $\leq$ 5 Mos.
Rolls Over (Back To Front)				By $\leq$ 5 Mos.
Sits Alone				By $\leq$ 8 Mos.
Moves From Laying To Sitting Unassisted				$\leq$ 10 Mos.
Weight-Bearing, Supported				
Walks Holding On				By $\leq$ 15 Mos.
Walks Independently				By $\leq$ 16 Mos.

MOTOR TESTING

	POWER			TONE			INVOLUNTARY MOVEMENTS*		
	Not Done	Normal	Abnormal	Not Done	Normal	Abnormal	Not Done	Normal	Abnormal
**Neck/Trunk** Muscles									
**Right Arm**									
Proximal									
Distal									
**Left Arm**									
Proximal									
Distal									
**Right Leg**									
Proximal									
Distal									
**Left Leg**									
Proximal									
Distal									

***Type of Involuntary Movements Seen**
Check all that are present

TYPE	?Present
Limb Tremor	
Choreoathetosis	
Dystonic Posturing	
Tics	

TENDON REFLEXES

TEST ITEMS		Not Done	Normal	Abnormal	Guidelines for Scoring
Biceps	Right				
	Left				
Brachioradialis	Right				
	Left				
Triceps	Right				
	Left				
Knee Jerk	Right				
	Left				
Quadriceps	Right				
	Left				
Ankle Jerk	Right				
	Left				
Babinski	Right				Upgoing toe is normal up to one year
	Left				
Elicited ankle clonus	Right				
	Left				

FINE MOTOR / COORDINATION

TEST ITEMS		Not Done	Normal	Abnormal	Guidelines for Scoring
Pincer Grasp	Right				Encourage to pick up small 2–3 mm. ball of rolled up paper
	Left				
Rapid Tap Index Finger	Right				Test from ~18 months: **"As fast as you can"** demonstrate index finger repetitively tapping with hands placed palms down on surface
	Left				
Reaching for object	Right				Observe for unusual or asymmetric tremor on reaching for object
	Left				
Sitting/ Standing Balance					

SENSORY

TEST ITEMS		Not Done	Normal	Abnormal	Guidelines for Scoring
Light Touch	Right				
	Left				
Pin Prick or Cold	Right				
	Left				

GAIT

Test Only if ≥ 12 months and walking without support

TEST ITEMS	Not Done	Normal	Abnormal	Guidelines for Scoring
Gait Walking				By ≥ 16 mos.
Gait Running				By 2 yrs of age

SCORING SHEET FOR PSOM-SNE

SUMARY OF IMPRESSIONS

After completing the PSOM-NE or equivalent detailed neurologic examination, summarize and grade your impressions in the following categories:

A. Sensorimotor Deficit (ANY motor or sensory abnormality including Cranial Nerve Deficits, Visual, and Hearing deficits)

	R side	L side
Not Done	n/t	n/t
None	0	0
Mild but no impact on function	0.5	0.5
Moderate with some functional limitations	1	1
Severe or Profound with missing function	2	2

Select the Sensorimotor Deficits You Observed (select all that apply)

☐ Global developmental delay ☐ Global hypotonia or hypertonia

☐ Hemiparesis ☐ Hemifacial weakness ☐ Hemiataxia ☐ Dysarthria ☐ Other Motor deficit

☐ Hemisensory deficit ☐ Other Sensory deficit

☐ Difficulty with vision

☐ Difficulty with drinking, chewing or swallowing

☐ Other, describe: ______________________________

B. Language Deficit – Production (including dysarthria)

Not Done	n/t
None	0
Mild but no impact on function	0.5
Moderate with some functional limitations	1
Severe or Profound with missing function	2

Describe the Language Production Deficits You Observed Here: ____

____

C. Language Deficit - Comprehension

Not Done	n/t
None	0
Mild but no impact on function	0.5
Moderate with some functional limitations	1
Severe or Profound with missing function	2

Describe The Language Comprehension You Observed Here: ____

____

D. Cognitive or Behavioural Deficit (specify which) ☐ **Cognitive** ☐ **Behavioural**

Not Done	n/t
None	0
Mild but no impact on function	0.5
Moderate with some functional limitations	1
Severe or Profound with missing function	2

Describe The Cognitive or Behavioural Deficits You Observed Here: ____

____

TOTAL SCORING: ___________/10

1. Have you/your child recovered completely from the stroke? ☐ Yes ☐ No
2. Does your child need extra help with day-to-day activities compared to other children their age? ☐ Yes ☐ No
3. Has the stroke affected you/your child's emotional state, behavior and feelings about his/herself? ☐ Yes ☐ No
   Does your child show any signs of depression? ☐ Yes ☐ No
4. Does the child use aids or assistive devices (e.g. splints, braces)? ☐ Yes ☐ No Specify:________________________
