# Supplementary material for: Inflammatory cytokines, placental pathology, and neurological outcomes in infants born to preterm preeclamptic mothers
Source: PLoS One. 2021 Nov 15;16(11):e0260094. doi: 10.1371/journal.pone.0260094 (PMC8592443; doi:10.1371/journal.pone.0260094)
Supplement: Attachment — Submitted filename: Response to reviewers.docx [file pone.0260094.s003.docx]

PONE-D-21-14686
Biomarkers of inflammation, placental pathology, and neurological outcomes in infants born to preeclamptic mothers
PLOS ONE

Dear Dr. Armstrong,

Thank you for submitting your manuscript to PLOS ONE. After careful consideration, we feel that it has merit but does not fully meet PLOS ONE’s publication criteria as it currently stands. Therefore, we invite you to submit a revised version of the manuscript that addresses the points raised during the review process.

If you can address the specific concerns detailed below, then we would be willing to reconsider a revised version. Before seeing and evaluating such changes, however, we cannot guarantee that your revised article would be accepted for publication. Should you decide to revise the manuscript for further consideration here, your revisions should address all points indicated by reviewers.

Please submit your revised manuscript within 30 days. If you will need more time than this to complete your revisions, please reply to this message or contact the journal office at [plosone@plos.org](mailto:plosone@plos.org). When you're ready to submit your revision, log on to <https://www.editorialmanager.com/pone/> and select the 'Submissions Needing Revision' folder to locate your manuscript file.

Please include the following items when submitting your revised manuscript:

- A rebuttal letter that responds to each point raised by the academic editor and reviewer(s). You should upload this letter as a separate file labeled 'Response to Reviewers'.
- A marked-up copy of your manuscript that highlights changes made to the original version. You should upload this as a separate file labeled 'Revised Manuscript with Track Changes'.
- An unmarked version of your revised paper without tracked changes. You should upload this as a separate file labeled 'Manuscript'.

If you would like to make changes to your financial disclosure, please include your updated statement in your cover letter. Guidelines for resubmitting your figure files are available below the reviewer comments at the end of this letter.

If applicable, we recommend that you deposit your laboratory protocols in protocols.io to enhance the reproducibility of your results. Protocols.io assigns your protocol its own identifier (DOI) so that it can be cited independently in the future. For instructions see: <http://journals.plos.org/plosone/s/submission-guidelines#loc-laboratory-protocols>. Additionally, PLOS ONE offers an option for publishing peer-reviewed Lab Protocol articles, which describe protocols hosted on protocols.io. Read more information on sharing protocols at <https://plos.org/protocols?utm_medium=editorial-email&utm_source=authorletters&utm_campaign=protocols>.

We look forward to receiving your revised manuscript.

Kind regards,

Claudio Romero Farias Marinho, Ph.D.
Academic Editor
PLOS ONE

Journal requirements:

When submitting your revision, we need you to address these additional requirements. ***We apologize, the manuscript was directly transferred from PLOS MEDICINE. We have addressed all of the journal requirements listed below 1-5.***

1. Please ensure that your manuscript meets PLOS ONE's style requirements, including those for file naming. The PLOS ONE style templates can be found at

<https://journals.plos.org/plosone/s/file?id=wjVg/PLOSOne_formatting_sample_main_body.pdf> and

<https://journals.plos.org/plosone/s/file?id=ba62/PLOSOne_formatting_sample_title_authors_affiliations.pdf>.

2. We note that you have stated that you will provide repository information for your data at acceptance. Should your manuscript be accepted for publication, we will hold it until you provide the relevant accession numbers or DOIs necessary to access your data. If you wish to make changes to your Data Availability statement, please describe these changes in your cover letter and we will update your Data Availability statement to reflect the information you provide. ***Please see Cover Letter.***

3. Please amend the manuscript submission data (via Edit Submission) to include authors : “Alexandra Sotiros MS , Dianne Thornhill PhD , Miriam D Post MD and Virginia D Winn MD “

4. Please amend your list of authors on the manuscript to ensure that each author is linked to an affiliation. Authors’ affiliations should reflect the institution where the work was done (if authors moved subsequently, you can also list the new affiliation stating “current affiliation:

   a. University of Colorado Anschutz Medical Campus (CU-Anschutz),

   b. Hemophilia and Thrombosis Center,

   c. Department of Pathology

   d. Stanford University School of Medicine, Department of OB/GYN,

   e. Departments of Pediatrics (Section of Neurology), Neurology, and OB/GYN (Basic Reproductive Sciences)

   f. Children’s Hospital Colorado.” as necessary).

5. Please ensure that you refer to Figure 2 in your text as, if accepted, production will need this reference to link the reader to the figure.

[Note: HTML markup is below. Please do not edit.]

Reviewers' comments:

Reviewer's Responses to Questions

**Comments to the Author**

1. Is the manuscript technically sound, and do the data support the conclusions?

The manuscript must describe a technically sound piece of scientific research with data that supports the conclusions. Experiments must have been conducted rigorously, with appropriate controls, replication, and sample sizes. The conclusions must be drawn appropriately based on the data presented.

Reviewer #1: Partly

Reviewer #2: Yes

2. Has the statistical analysis been performed appropriately and rigorously?

Reviewer #1: Yes

Reviewer #2: Yes

3. Have the authors made all data underlying the findings in their manuscript fully available?

The [PLOS Data policy](http://www.plosone.org/static/policies.action#sharing) requires authors to make all data underlying the findings described in their manuscript fully available without restriction, with rare exception (please refer to the Data Availability Statement in the manuscript PDF file). The data should be provided as part of the manuscript or its supporting information, or deposited to a public repository. For example, in addition to summary statistics, the data points behind means, medians and variance measures should be available. If there are restrictions on publicly sharing data—e.g. participant privacy or use of data from a third party—those must be specified.

Reviewer #1: Yes

Reviewer #2: No

4. Is the manuscript presented in an intelligible fashion and written in standard English?

PLOS ONE does not copyedit accepted manuscripts, so the language in submitted articles must be clear, correct, and unambiguous. Any typographical or grammatical errors should be corrected at revision, so please note any specific errors here.

Reviewer #1: Yes

Reviewer #2: Yes

5. Review Comments to the Author

Please use the space provided to explain your answers to the questions above. You may also include additional comments for the author, including concerns about dual publication, research ethics, or publication ethics. (Please upload your review as an attachment if it exceeds 20,000 characters)

Reviewer #1: Thank you for the opportunity to read this article. The study provides interesting information about the association of inflammatory cytokines, placental injury, and neurological outcomes in newborns of pre-eclamptic pregnant women. Objectives of this study are well stated. Many important details of the methods, especially of sampling from the participants, are omitted. I expect that previous publications provide this information, but readers of this article should not have to seek out those papers in order to understand the study. Thus, it needs several major and minor changes before it can be published.

General comments:
- There are some sections that need English language editing - it would be helpful if the manuscript were re-reviewed with attention. ***All authors are native English speakers. We have done a major re-write. We are hopeful that the reviewers will find the manuscript flows better and is grammatically correct.***

- I suggest changing the title, because the use of the word “biomarker” refers to the idea that the authors tested the hypothesis of these cytokines to predict some adverse event, which is not true. For example: “Inflammatory cytokines, placental pathology, and neurological outcomes in infants born to preeclamptic mothers.” ***Thank you for the suggestion, we have revised the title****.*
- Consider revising all abbreviations in the text, as some are mentioned without a description. The excessive use of abbreviations makes it difficult to read and understand the entire text. Authors should use abbreviations only when necessary. Following the guidelines of the journal: 1- Do not use non-standard abbreviations unless they appear at least three times in the text; 2- Keep abbreviations to a minimum. ***Done.***
- I strongly suggest that the authors write the article following the guidelines of STROBE, attaching the checklist as supplementary material. ***Included S2_File***

Methods
- It would be helpful to know (briefly) how the women in this study were recruited - inclusion/exclusion criteria must be well defined; when were pregnant women recruited - at delivery? ***Clarified, Line 88-98.***
- Line 77-78: The authors stated that pre-eclampsia was identified according to standard practice. What standard practice? WHO? Missing reference. ***Clarified – UCHealth Obstetrics standard practice, requirements in text Line 91-97.***
- Line 84-85: Could the authors comment on why multiple pregnancies were included in the analyzes? Wouldn't the chances of these fetuses develop neurological problems be greater, causing a bias in the analysis? ***Yes, we agree that twin gestation has the potential for worse outcome. Our statistician Dr. Thornhill reran the statistics with and without the twin gestations and has ensured that the outcomes and statistical analysis remained the same. Because of our small number and no change to statistical analysis, we kept the twin gestations in our cohort.***
- Line 104: Were the professionals blinded to the placental analyzes? ***Yes, and clarified Line 140-141***
- It is very important to note that several sections of the study, but especially “Cytokine Analysis” and “Postnatal evaluation” are written identically to the previous study of the group (ref. 31), without even mentioning that this had already been done before. I don't believe this is correct! I suggest that the authors cite previous studies. ***The PSOM has not been utilized in the preterm/preeclampsia cohort in conjunction with cytokine and placental analysis in any other studies aside from our group, which we have now cited in the methods. We have included the PSOM as supplemental material for the reader S1_File.***
- How was gestational age ascertained? This is important because the authors use the gestational age of 32 weeks for cut-off. ***Clarified Line 104.***
- Line 134: The “Statistical Analysis” section should be better written - data on the adopted confidence interval, p-value, are missing. I suggest that the authors read the journal's guidelines: section “Reporting of statistical methods”. ***Dr. Thorne, our statistician, has rewritten and clarified this section.***
- Line 137-139: The authors stated that: “Gestational age at delivery was dichotomized into ≤32 weeks and >32 weeks for paired comparisons to maintain comparative consistency with our previous study cohorts (30, 31)”. However, this does not seem to me to be a plausible justification. A more biological/clinical justification is needed. Could the authors comment? ***Line 151-153, references also added***.

Results
- Line 153-159: The authors present the results of all the cytokines evaluated, but Table 2 shows only two (IL-6 and IL-8). I suggest adding the entire result in the table, together with the p-value and the description in the legend of the statistic used. ***We agree and have redone Table 2.***
- In addition to the previous comment, tables 1, 2 and 3 need adjustments. The tables must have appropriate titles and legends, with a description of any abbreviation, and the statistics used; and the p-value must be indicated in the table. Authors should read the journal's guidelines: 1- Place each table in your manuscript file directly after the paragraph in which it is first cited (read order); 2- Tables require a label (e.g., “Table 1”) and brief descriptive title to be placed above the table. Place legends, footnotes, and other text below the table. ***We apologize, our manuscript was directly transferred from PLOS Medicine which was formatted differently. We have fixed all Tables and Figures per PLOS ONE guidelines.***
- Line 160-164: I did not understand why the authors describe these results only for males. Could you explain? ***Clarified Line 158-160***
- Line 181-184: The way these results are presented seems to me confused. I suggest that the authors present a table or figure that contains these results, with a complete description of the statistical method used. ***We agree that the results section did not flow well. We have significantly revised and rewritten the Results section as well as redone our tables.***
- Line 187: Figure 1 or 2? ***Corrected.***

Discussion
- Line 227-229: Only corroborating with a previous comment, the authors presented results only for male newborns and in this paragraph of the discussion they describe only about female newborns. If the authors consider gender to be important, both must have results and be discussed***. Expanded Discussion, Line 295-303.***
- The authors presented results on parity, diseases such as chronic hypertension and diabetes, type of delivery (Table 1) and did not discuss anything about it. Could you comment since the N of these variables is considerable? ***Line 234-235. We are limited by our small numbers, and suspect that a larger cohort may bear out more of these effects.***
- The authors divided their population according to their gestational age (greater or less than 32 weeks) and did not discuss anything about it. This is a key point of the study that must be discussed. ***Agree - added Line 284-293***

Reviewer #2: The manuscript “Biomarkers of inflammation, placental pathology, and neurological outcomes in infants born to preeclamptic mothers” by Armstrong et al, analyzed serum inflammatory markers of preeclampsia and aimed to correlate these markers and placental inflammation to neonate brain injury. The manuscript presents findings that corroborate the presence of placental inflammation and brain injury in newborns, which was correlated to gestational age less than 32 weeks. However, this reviewer believes some points need to be better explained and added to improve the paper.
\# Major limitations
Material and methods
In general, the methodology was appropriated to collect the data and perform the intended analysis. According to this reviewer, the topic of the postnatal evaluation is not clear. Authors should provide more details and make clearer the information about the parameters used to determine the scores to classify the brain injury.
Results
This reviewer recommends that the following points are addressed:
1. Line 160 provides information on the percentage of male neonates with GAD < 32 weeks but there is no explanation/discussion about the relation of these observations with the pathology. Please add a statement on the manuscript to clarify the relevance of the above mentioned data. ***Clarified Line 158-160***
2. In the paragraph between the lines 166-171: It was not clear for this reviewer if the analysis was performed with the placentas from GAD > 32 weeks or if both data from GAD <32 weeks and GAD > 32 weeks were considered in this analysis. Please clarify this information. ***Dr. Thorne, our statistician, has rewritten and clarified this section.***
3. Line 187: Figure 1 was cited following the sentence “Within that sample at the 6-month visit (Figure 1)”. This reviewer does not understand the relationship between the neurological outcome and Figure 1, if there is any. Figure 1A represents chronic villitis and figure 1B is a figure of an umbilical artery. Please clarify the intended meaning. ***We apologize for the confusion. This has been corrected. We have removed Figure 1 Placental pathology as we felt it did not add to our paper. The new Figure 1 should be and is neurological outcome correlated with gestational age. This has been corrected and uploaded at Fig 1.***
4. Figure 1: As highlighted by the authors, matching healthy controls in a limitation of this study. Nevertheless, this reviewer believes that evidence of normal placental structure and umbilical artery should also be provided. ***Figure 1 Placental Pathology has been removed as we felt that it did not add to and possibly detracted from the paper; there is not a true “normal healthy” preterm placenta standard***
5. Regarding clinical and experimental database. This reviewer believes that in the interest of data availability, authors should make all data underlying the described finding available in the form of supplementary material. ***I spoke to our IRB director (I am also an IRB Chair) about this given during study enrollment time frame public data repositories were not mandated. Our participants did not give consent for this public availability, and the nature of our research/variables as medical records plus the risk of identifiability violate our research ethics policy. I cannot feasibly reconsent our participants for this. I would be allowed to release data on a case-by-case request to me as the corresponding author if the request if reasonable and with permission from our IRB. I also emailed the editors separately about this and have not been told this precludes publication.***

Specific comments for figures and tables
Table 1. Magnesium levels are represented in table 1but are not mentioned in the text. Please explain the importance to measure magnesium for this analysis. ***Clarified Line 170-171***
Table 2. The table displayed information regarding the IL-6, IL-8, but no information about TNF-alpha levels. This reviewer suggests including this information. ***Agree, Table 2 is revised to include all cytokines***
Figure 1. Chronic villitis (A) and fetal inflammatory response (B). Authors should have used arrows signalizing the observed evidence observed. Please provide a new version of Figure 1. ***This figure was removed in the revision.***
Figure 2 was not mentioned in the text. Authors should explain its results and discuss its relevance in the text. ***Our apologies, this has been rectified (see above).***
Discussion
In the study, the results showed an association between gestational age at delivery ≤ 32 weeks and worse neurological outcomes at 6-month CGA. There is no association between maternal or fetal cytokine activity and neurological outcomes, even in the samples with evidence of maternal malperfusion in the placenta. The authors discuss the current methodology (cranial ultrasound) used to predict neurological function, suggesting that the injury could be at a molecular level, being the ultrasound not appropriated to predict neurological dysfunctions. This reviewer agrees, but alternative methods should be provided by the authors to fulfill this shortcome. For example, but not limited to, is the study of Setänen et al., 2016 (doi.org/10.1111/dmcn.13030) used volumetric neonatal magnetic resonance imaging to predict the neuromotor outcome in infants born preterm. ***Expanded, Line 304-325.***

\# Minor limitations
Line 167: Chronic villitis in Figure 1A. ***This figure has been removed.***
