# Supplementary material for: Inflammatory cytokines, placental pathology, and neurological outcomes in infants born to preterm preeclamptic mothers
Source: PLoS One. 2021 Nov 15;16(11):e0260094. doi: 10.1371/journal.pone.0260094 (PMC8592443; doi:10.1371/journal.pone.0260094)
Supplement: Attachment — Submitted filename: Response to reviewers_Revision 2.docx [file pone.0260094.s004.docx]

PONE-D-21-14686

**Per the editor: “Your changes to the manuscript has been re-reviewed by the original reviewers.  Reviewer No. 1 has made some suggestions that should strengthen the manuscript.  Please respond to Reviewer No. 1's suggestions.”**

**Reviewer #1:** Armstrong et al did a good job of reviewing the article. The authors have an interesting data set with potential for an interesting manuscript. However, some minor revisions still need to be resolved before publication.

INTRODUCTION
• Line 54-60: The word preeclampsia is used six times in this paragraph. Consider using synonyms.

- Agree. This paragraph has been edited.
• Review the citations made in the introduction. A few sentences have no citation.

- We have edited and confirmed all appropriate citations.
METHODS
• Line 105-106: The accuracy of gestational age (GA) is important. So, ultrasound in the first trimester of gestation can represent a better tool to access GA. Did the authors have that information? This can be added as a limitation of the study.

-This has been added as a limitation in lines 341-343. Given our Labor and Delivery population (different arenas of prenatal care) and clinical data collection methods, these data were not available.
• Line 141: Considering the complexity of neurological assessment, was the examiner trained or specialized? Consider providing this information.

- Dr. Armstrong is a known specialized expert in neonatal and vascular neurology. This has been noted in the methods.
• The Pediatric Stroke Outcome Measure was designed to assess outcomes after stroke in infants and children. It would be interesting to include a justification for using this assessment tool.

- Added and PSOM included in Supplemental Material.
RESULTS
• Lines 89/91 and 167: In the methods section, the age group is described as 18-50 years and gestational age <37 weeks. However, in table 1 the age group is from 18 to 45 years old, and the gestational age is up to 39.4 weeks. Could the authors check it out?

- These numbers are correct. Per the methods study inclusion criteria were women 18-50 years old, while Table 1 presents the age range of the women actually consented into the study, with the oldest being 45 years old. As for gestational age, per the methods study inclusion criteria was gestational age <37 weeks at time of hospitalization for preeclampsia, and Table 1 provided gestational age at time of delivery. This has been clarified in the methods and Table 1.

• Line 174: In the text it shows the Hispanic ethnicity at almost 40% (26/67), but in the table it is 34% (26/76). Could the authors also check? In addition, as Table 1 describes the study population, I suggest including the other ethnic groups.

- There were no other ethnicities in our study population. This is representative of the Rocky Mountain Region, which is mainly a white population non-Hispanic population. Table 1 and text explicitly updated regarding this.
• Line 224 - Table 5 is missing.

-Thank you for catching this. We had removed this Table and instead presented as text per the recommends at the prior revision.
• Lines 239-241: The adjusted values shown in this part do not seem close to significance as stated by the authors. Consider reviewing this sentence.

-Agree, we deleted the sentence pertaining to IL-6 as it does not approach significance.
GENERAL
• Consider adding legend to all tables as appropriate.

-Thank you, we have discussed and have no additions for legends in the Tables. We have added additional legend information to Fig 1.
• Line 355-358: I guess this paragraph is misplaced.

-Thank you, yes this was improperly inserted as has been removed.
• Figure 1 is distorted. Perhaps it could be enhanced to improve the visualization by the readers.

-We have tried to enhance the Figure to the best of our ability and within the limitations of the software. If the reviewer clicks on the direct link in the constructed pdf for the uploaded .tiff Figure, the Figure is clear. It appears to be a glitch with the pdf conversion that we do not know how to fix on the author submission end.
